# Posture and Virtual Reality: How a Head-Mounted Display Affects Postural Stability and Plantar Pressure Parameters in Healthy Population

**DOI:** 10.3390/jfmk11030247

**Published:** 2026-06-25

**Authors:** Ciro Ivan De Girolamo, Allegra Fullin, Ersilia Vallefuoco, Annunziata Attena, Angela Lucariello, Pasquale Arpaia, Paolo De Blasiis

**Affiliations:** 1 Department of Advanced Biomedical Sciences, University of Naples Federico II, 80131 Naples, Italy; ciroivan.degirolamo@unina.it (C.I.D.G.); allegrafullin92@hotmail.it (A.F.); 2 Department of Electrical Engineering and Information Technology (DIETI), University of Naples Federico II, 80131 Naples, Italy; ersilia.vallefuoco@unina.it (E.V.); nancyattena97@gmail.com (A.A.); pasquale.arpaia@unina.it (P.A.); 3 Department of Sport Sciences and Wellness, University of Naples “Parthenope”, 80100 Naples, Italy; angela.lucariello@uniparthenope.it; 4 Department of Health Sciences, University of Basilicata, 85100 Potenza, Italy

**Keywords:** posture, virtual reality, head-mounted display, baropodometry, stabilometry

## Abstract

**Background**: The Postural Control System is affected by sensory inputs in stabilizing posture. The impact of postural receptors can be quantitatively evaluated by baropodometry. The lack of a standardized testing environment can decrease the reliability of baropodometric results. Virtual reality (VR) might represent a useful standardization tool. This study aimed to investigate the effects of virtual environment on plantar pressure and postural stability parameters by using a Head-Mounted Display (HMD). **Methods**: 50 healthy young adults underwent a baropodometric exam in upright standing under four conditions: Open Eyes (OE), Closed Eyes (CE), open (HMD-OE) and closed eyes (HMD-CE) conditions while wearing an HMD. **Results**: a negligible effect of VR on intrasubject variability of plantar pressure and stabilometric parameters. Moreover, no significant differences in the latter ones were found between OE conditions without and with headset (OE vs. HMD-OE), highlighting no impact of VR; instead, a significant increase in body sway was found in the closed eyes condition compared to OE ones with and without headset (CE vs. OE, CE vs. HMD-OE), underlining the effect of visual deprivation, whereas no significant difference was observed between the HMD-CE and OE conditions and a significant decrease in HMD-CE compared to the CE condition, showing the sensory-proprioceptive effect of the HMD. Eventually, no significant differences in plantar pressure parameters were generally found in different conditions. **Conclusions**: These findings highlighted the specific effect of visual afferents differently from proprioceptive ones by headset use and the absence of the VR impact on postural stability, suggesting a possible role of virtual reality in standardizing instrumental postural exam.

## 1. Introduction

Posture refers to the overall position of the body segments in space and to the joint alignment, maintained in balance through a complex and precise nervous system regulation producing continuous and adaptative neuromuscular strategies [1]. The Postural Control System (PCS), formed by a network of neural structures within the Central Nervous System and peripheral sensory receptors, processes visual, vestibular, stomatognathic and somatosensory afferents [2,3] and elaborates motor outputs modulating myofascial chains in order to stabilize the skeletal structure against gravity [4]. In this context, the PCS can be considered a dynamic sensorimotor process, in which the Central Nervous System continuously integrates multisensory information and adjusts multi-joint motor responses to preserve body orientation and equilibrium [5]. Therefore, the PCS plays a key role in maintaining upright posture during interaction with the surrounding environment whether in static or in dynamic conditions [6,7], even the most unstable ones [8]. Posture is widely evaluated using clinical and instrumental methods, such as X-rays and 3D motion analysis, which are considered the gold standards for assessing static [9] and dynamic posture in both healthy and pathological populations [10,11,12]. Moreover, baropodometric and stabilometric examinations are commonly used to evaluate plantar pressure and postural stability parameters, respectively [13,14,15]. In particular, baropodometry is an electronic mat, composed of capacitive or resistive sensors, and is also able to assess the foot-to-ground load distribution by mapping the pressure in different plantar regions [13], whereas stabilometry is a force platform that evaluates the body’s oscillations by analyzing the displacement of the body’s Center of Pressure in upright standing during different conditions in which specific sensory receptors can be tested [14,15]. Among these receptors, the visual system provides an important contribution in controlling postural stability, even if the latter depends on the integration and context-dependent reweighting of visual, vestibular, and somatosensory inputs, according to both environmental conditions and sensory or mechanical constraints [16,17,18,19]. Several studies have quantitatively investigated the role of visual receptors in healthy subjects [20], and the impact of different distances from the viewing target [21] and of the type of lighting and colors of the external environment [22] on the stabilometric parameters. Finally, the influence of the sound on the postural control was also assessed in static [23] and dynamic conditions [24].

These previous studies highlighted the need to standardize the room where a posture exam is performed, recommending white walls, a standardized target distance, a level floor and a silent environment [13,20,21]. A possible technological solution could be the use of virtual reality (VR) [25,26,27,28,29,30,31,32,33,34,35] that reproduces an ideal room such as a controlled, immersive, and realistic environment in which users can exhibit behaviors similar to real-life scenarios [36]. This capability, combined with its potential to ensure reproducibility and precisely control sensory input, underscores the value of VR as a flexible and ecologically valid tool for motor assessment [37]. The virtual immersive experience is typically achieved through head-mounted displays (HMDs), which provide multi-sensory input and enable users to explore virtual environments, whether real or imaginary, while sitting comfortably at home or during a postural assessment [32,38].

Previous studies [25,26,32,39,40] evaluated how VR affects postural stability in healthy adults in both open and closed eyes conditions. In particular, VR-HMD use may induce sensory conflict due to visual–vestibular mismatch [41], requiring sensory reweighting processes that can alter postural responses and, in some cases, contribute to cybersickness-related symptoms [42]. Nevertheless, such symptoms are observed in healthy subjects who underwent virtual dynamic images during quiet standing [43,44] and challenging static or dynamic motor tasks [27,45], while they were not reported in the virtual static environment during quiet standing [27]. In fact, no significant effect on static balance, but only during dynamic performances, was found in a physical environment with respect to a virtual one viewed through an HMD [27]. Moreover, a negligible effect of HMD mass on postural stability was found [26,27,45], while one related to the control of trigeminal sensory pathways on posture should be explored in greater depth [46].

To the best of our knowledge, no study has systematically and thoroughly investigated the changes and variability in plantar pressure and postural stability parameters induced by the use of immersive VR during quiet bipedal standing. Specifically, the Coefficient of Variation in baropodometric and stabilometric parameters, the impact of the HMD mass on plantar pressure parameters, and intra- and inter-foot comparisons among plantar regions assessed in a virtual environment during different visual conditions still remained unexplored. Moreover, the present study also aimed to verify how a standardized virtual room may affect the abovementioned parameters, suggesting the idea of a reproducible setting for instrumental evaluation identically replicable across all laboratories in order to minimize variability due to differing physical testing conditions.

Therefore, this study addresses two main research questions (RQs):

RQ1: Does the use of an ideal and standardized virtual reality room during baropodometric assessment affect plantar pressure and stabilometric parameters, as well as their intra-subject variability across different testing conditions?

RQ2: Does wearing a head-mounted display (HMD) influence plantar pressure and stabilometric parameters, particularly due to the effect of its mass and sensory stimulus?

## 2. Materials and Methods

### 2.1. Subject Population

The sample size was determined a priori using analysis using G*Power software (version 3.1.9.7) for a repeated measures ANOVA with within-subject factors (effect size f = 0.25, α = 0.05, β = 0.95, number of measurements = 4, number of groups = 1, corrections among repeat measures c = 0.5, non-sphericity correction ε = 1). Since no previous studies were available with the same design and the same combination of stabilometric and plantar pressure outcomes, a medium effect size (f = 0.25) was adopted, the correlation among repeated measures was set at 0.50 as an intermediate assumption, and the non-sphericity correction was initially set to ε = 1 for sample size planning purposes. This a priori analysis provided a total sample size of at least 36 participants. Therefore, 50 healthy participants (M = 32, F = 18) were enrolled, ensuring a sample size larger than the minimum estimated requirement.

The sample presented a mean age of 21.72 ± 2.02 years, a mean body weight of 68.6 ± 11.77 kg, a mean height of 1.72 ± 0.09 m, and a mean body mass index (BMI) of 22.60 ± 2.14 kg/m^2^. Participants were recruited from the University of Federico II and University of Basilicata through advertisements distributed among students and academic colleagues. Inclusion criteria were: healthy young adults aged 18 to 30 years, BMI between 20 and 25 kg/m^2^, right-limb dominance, determined by asking each subject which leg they would use to kick a ball as far as possible. The inclusion of only right-limb-dominant participants was intended to reduce the potential influence of limb dominance on plantar pressure distribution and stabilometric outcomes, thereby improving data consistency and limiting inter-subject variability. Exclusion criteria: no pain; no surgery within the past 6 months; no musculoskeletal injury in the past 3 months; no dental surgery or use of dental implants; no prostheses or corrective orthoses; no neurological or visual disorders; no skeletal dysmorphism; no cognitive impairment; no competitive practice of asymmetric sports [47].

The study was carried out according to the Helsinki Declaration and written informed consent was obtained from all patients participating in this study.

### 2.2. VR Application

The VR application was developed using Unity (version 2022.3.58f1) and specifically released for Meta Quest 2 (mass = 503 g). The virtual environment was designed to simulate an idealized laboratory, providing a neutral space for the postural assessment. The scene consists of a completely white empty room, devoid of furniture or shadows. This minimalist design ensures uniform lighting and eliminates visual distractions, helping participants focus on the task [21,48]. The environment was built to real-world scale (1:1) to enhance perceptual realism and ensure consistency between physical movements and their virtual representation [32]. Similarly, the dimension and position of the visual target, in terms of both distance from the user, position of the target and its height, were replicated in the VR scenario to maintain spatial consistency between the real-world and virtual tasks.

### 2.3. Procedure

Participants were evaluated in upright bipedal posture with the arms relaxed along the body close to the thighs and the head in neutral position using a resistive-sensor pressure plate (P-Walk FM12050, BTS-Bioengineering, Milan, Italy; 200 × 50 cm, 10,000 sensors/m^2^; sampling rate 50 Hz). The accuracy and repeatability of this pressure plate for static measurements have been previously validated by comparison with a precision scale and Bland–Altman analysis [14].

The procedure was divided into four sessions, during which each participant completed twelve 30 s stabilometric examinations. Each session was carried out in randomized order with or without the use of the head-mounted display (HMD) and under both open (OE) and closed (CE) eyes conditions. Randomization was performed using an online Google coin-flip tool, where “heads” indicated the HMD condition and “tails” the non-HMD condition; a second coin flip determined the eye condition, with “heads” corresponding to OE and “tails” to CE.

Before the experimental trials using the HMD, a 3 min familiarization period was provided to minimize possible adaptation effects on postural responses.

**Figure 1:**
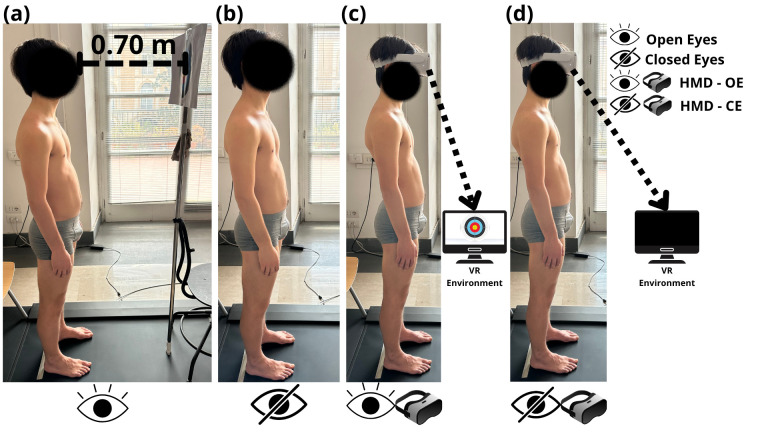
Healthy young adult assessed in upright posture during four conditions: (**a**) OE (Open Eyes), while viewing a target placed 0.70 m in front of them in real environment (non-HMD); (**b**) CE (Closed Eyes) with closed eyes without HMD; (**c**) HMD-OE (Head-Mounted Display with Open Eyes) while wearing a Head-Mounted Display that shows a virtual environment with a visual target placed at 0.70 m; (**d**) HMD-CE (Head-Mounted Display with Closed Eyes) while wearing a Head-Mounted Display with closed eyes.

In particular, two sessions of three trials were conducted with a real visual target placed 0.7 m from the subject’s heels, while the other two sessions were performed using a head-mounted display (HMD) to immerse the subject in a virtual environment replicating the ideal room, with the reproduced target placed at the same distance. The choice of the target distance was made in a range set in accordance with the criteria reported in our previous study [21], in which the 0.7 m distance was chosen, because 0.4 m is defined as the minimum distance below which the beginning of the blur occurs and because the measurement was taken from the heels rather than directly from the eyes. In fact, given the individual variability in the distance from the heels to the eyes, the actual eye-to-target distance was expected to be less than 0.7 m but still above 0.4 m. The center of the target was positioned directly ahead of the subject, at a 0° visual angle relative to the horizontal plane and adjusted to each individual’s height using an adjustable rod. The target featured concentric colored circles within a white rectangle measuring 29.7 cm by 42 cm. Participants were asked to adopt a self-selected, comfortable foot position in order to decrease variability of stabilometric parameters [49]; the feet placement was maintained consistently throughout each session (Figure 1). During the trials, subjects remained seated in a chair placed behind them for keeping their foot placement unchanged between acquisitions. Moreover, the participants were instructed to stand as still as possible, breathe normally, and avoid unnecessary movements. In open eyes conditions, they fixated on the target placed in front of them, whereas in closed eyes conditions they kept their eyes gently closed. Trials were considered valid if posture was maintained without stepping, loss of balance, or marked movement artifacts; otherwise, the trial was repeated.

### 2.4. Data Acquisition and Statistical Analysis

The pressure plate was calibrated before each acquisition; weight calibration was performed by entering the participant’s body weight into the software. The following clinically relevant stabilometric and plantar pressure parameters, described in a previous study [15], were exported by P-Walk software (P-Walk FM12050 BTS-Bioengineering, Milan, Italy); without applying additional custom filtering: Center-of-Pressure sway area (CoPsa; mm^2^); Length Surface Function (LSF; mm^−1^); Center-of Pressure speed (CP speed; mm/s); total foot (Tf), rearfoot (Rf), midfoot (Mf), and forefoot (Ff) loads (%); the foot contact area (FCA; cm^2^); and Arch Index (AI; %); mean and peak pressures (Pmean and Pmax; kPa) of total foot. In addition, Regional Contact Areas (RCAs), and mean and peak pressures of Ff, Mf and Rf were calculated in post-processing data using an ad hoc algorithm. All plantar pressure parameters were calculated for both left (l) and right sides. Load parameters were normalized to body weight (%BW). Mean pressure parameters at Tf, Rf, Mf and Ff were calculated as the ratio between weight and associated area. In addition, according to previous studies [14,15,21], for each parameter, intra-subject (inter-trial) variability was assessed via the Coefficient of Variation (CV) across 3 trials for all conditions (OE, CE, HMD-OE and HMD-CE).

Statistical significance was set at *p* ≤ 0.05. The Shapiro–Wilk and Mauchly tests were used to assess the assumptions, respectively, of normality and sphericity for all the variables of interest; if the latter assumption was not satisfied, the Greenhouse–Geisser correction was applied. According to the type of distribution (parametric or non-parametric), mean with Standard Deviation (Mean ± SD) and range (minimum, maximum) or median with 25th–75th percentiles were used to report all values.

For inferential analyses across conditions, trial-level data were first summarized ((Mean ± SD) and range (minimum, maximum) or median with 25th–75th percentile) into a single participant-level value for each parameter and condition, so that repeated trials were not treated as independent observations.

Inter-condition comparisons for CVs of all parameters across the four conditions (OE, CE, HMD-OE, and HMD-CE) were performed using parametric repeated-measures ANOVA or the non-parametric Friedman test for both sides. The same statistical procedures were applied to test significant differences across conditions for stabilometric parameters. For plantar pressure parameters, both intra- and inter-condition analyses were conducted. First, a two-way ANOVA or Friedman test (Factor 1 = foot side; Factor 2 = experimental condition) was used for plantar pressure variables (Load, Pmean, FCA and Pmax) of the total foot and for AI, to assess inter-foot (right vs. left) differences within each experimental condition and across all conditions. Second, repeated-measures ANOVA or the Friedman test (on both sides) was used to assess for each foot inter-region (Ff, Mf and Rf) comparisons within each condition and intra-region comparisons across conditions. When ANOVA/Friedman indicated significant effects (*p* ≤ 0.05), post hoc analyses with Tukey/Durbin–Conover correction were applied to identify specific pairwise differences.

Statistical analysis on collected data was performed by Jamovi (version 2.3.18) and R Statistics (version 4.3.2).

## 3. Results

### 3.1. Effect of Visual Conditions, Virtual Reality and Head-Mounted Display Use on Variability of Plantar Pressure and Stabilometric Parameters

**Figure 2:**
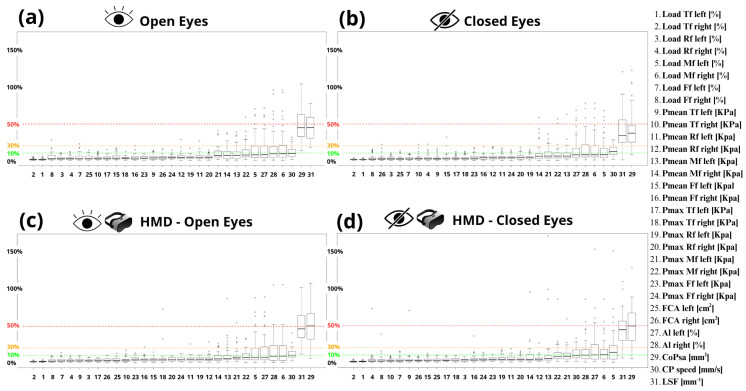
Intra-subject (inter-trial) variability of stabilometric and plantar pressure parameters represented via boxplots (median, 25th–75th percentiles) of Coefficient of Variation (CV) for each experimental condition. CVs in percentage for all parameters are sorted in ascending order according to the median values. Figure Note: Total foot (Tf), rearfoot (Rf), midfoot (Mf), forefoot (Ff), mean pressure (Pmean), maximum pressure (Pmax), Foot Contact Area (FCA), Arch Index (AI), Center of Pressure (CoP), Center-of-Pressure sway area (CoPsa), Center-of-Pressure speed (CP speed), Length Surface Function (LSF), (**a**) OE (Open Eyes); (**b**) CE (Closed Eyes); (**c**) HMD-OE (Head-Mounted Display with Open Eyes); (**d**) HMD-CE (Head-Mounted Display with Closed Eyes). The green, orange, and red dashed lines indicate, respectively, the thresholds of 10%, 30% and 50%.

Figure 2 is reporting the boxplots of the intra-subject Coefficient of Variation (CV) and their inter-subject distribution for each parameter. CVs of all 31 parameters were analyzed independently. These CVs are sorted in ascending order by the median value. The CV thresholds of 10%, 30%, and 50% were used only for descriptive purposes and were adopted in agreement with previous studies on stabilometric and plantar pressure variability using similar protocols [14,21,49]. The largest variability (30% < median CV < 50%) was found for the Center-of-Pressure sway area and Length Surface Function in all conditions, while Center-of-Pressure speed and all plantar pressure parameters showed a low variability (median CV < 10%) as reported in Figure 2. The only exception was found in the CE and HMD-CE conditions: specifically, in the CE condition (Figure 2b), CP-speed showed slightly higher variability (10% < median CV < 30%), while in the HMD-CE condition (Figure 2d), a similar increase in variability was observed for the left midfoot load.

The multiple comparison implemented via repeated-measures ANOVA or Friedman test across four conditions showed differences only for the following plantar pressure parameter: load in midfoot region on the left side (*p* = 0.044) (Table 1a). In particular, results of post hoc tests corrected for multiple comparisons with the Tukey method (Table 1b) showed a significant increase for CV of load midfoot left in the HMD-CE condition with respect to the OE one (*p* = 0.013). No significant difference was found for CVs of stabilometric parameters. However, trends were observed for CoPsa (*p* = 0.058) and LSF (*p* = 0.071).

### 3.2. Effects of Visual Conditions, Virtual Reality and Head-Mounted Display Use on Postural Stability Parameters

Regarding the effect of different experimental conditions on stabilometric parameters, statistical analysis across the multiple comparisons (Table 2a) highlighted significant differences for CoPsa (*p* = 0.029) and LSF (*p* = 0.013). In particular, results of post hoc tests corrected for multiple comparisons with the Tukey method (Table 2b) showed: a significant increase in CoPsa and a significant decrease in LSF in the CE condition compared to both OE conditions without (CE vs. OE; *p* = 0.008 for CoPsa and *p* = 0.003 for LSF) and with headset (CE vs. HMD-OE; *p* = 0.010 for CoPsa and *p* = 0.006 for LSF), while the post hoc comparison between CE and HMD-CE for CoPsa showed a borderline difference, which should be interpreted cautiously (*p* = 0.05); moreover, no significant differences were found between CE conditions with headset compared to the OE one with and without headset (HMD-CE vs. OE, HMD-CE vs. HMD-OE) and between OE conditions with and without headset (OE vs. HMD-OE).

### 3.3. Effects of Visual Conditions, Virtual Reality and Head-Mounted Display Use on Plantar Pressure Parameters

Regarding the statistical analysis of the plantar pressure parameters, it was necessary to perform both intra- and inter-condition comparisons for each foot. Specifically, analyses were conducted to: (Section 3.3.1) assess differences between left and right total foot parameters within each experimental condition and across the different conditions; (Section 3.3.2) examine the distribution and configuration of plantar pressure within the three predefined foot regions for each condition and across conditions. This approach allowed us to comprehensively characterize both foot-specific and condition-specific variations in plantar loading patterns.

#### 3.3.1. Comparison of Total Foot Parameters Between Left and Right Side Within Each Experimental Condition and for Each Foot Across Different Conditions

Inter-foot (left vs. right) comparison was implemented for each condition (intra-condition) and across different experimental conditions (inter-conditions) via two sides ANOVA for plantar pressure parameter of total foot as reported in Table 3. In particular, results of intra-condition analysis revealed no significant difference between left and right sides for load of Total foot and Arch Index; instead, a significant increase in FCA (<0.001) and significant decrease for Pmean and Pmax of Total foot were found on the right side with respect to the left one in each condition, except for Pmean in the HMD-CE condition. Moreover, regarding the inter-conditions analysis of all total foot parameters for each side, Table 3 showed no significant effect of visual conditions, virtual reality and Head-Mounted Display use.

#### 3.3.2. Comparisons of Foot Regions (Rear-, Mid-, Forefoot) for Each Side Within Experimental Condition and of Each Foot Region for Each Side Across All Conditions

**Figure 3:**
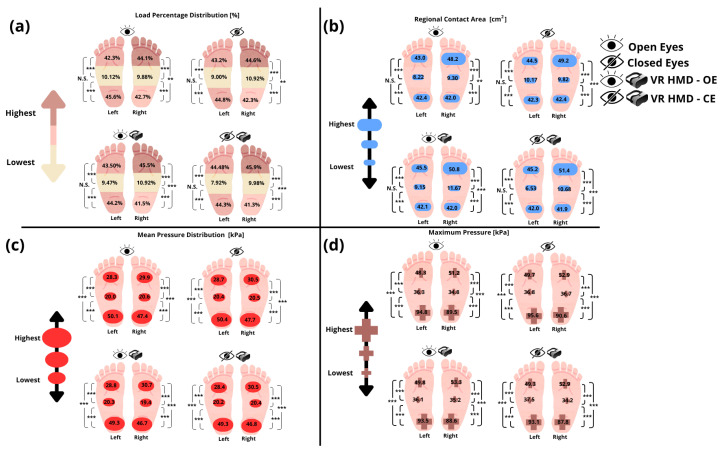
Graphical representation of the plantar footprint, illustrating the values of three foot regions for the following plantar pressure variables: (**a**) load distribution [%], (**b**) mean pressure [kPa], and (**c**) maximum pressure [kPa], (**d**) Regional Contact Area [cm^2^]. Figure note: The asterisks indicate statistically significant differences between regions and between feet, with 0.001 < *p* < 0.01 (**) and *p* < 0.001 (***); no statistically significant difference (N.S.); Open Eyes (OE); Closed Eyes (CE), virtual reality (VR), Head-Mounted Display (HMD).

Results of inter-region intra-foot intra-condition analysis showed significant differences (*p* < 0.001) for Pmax and Pmean values across three regions of the foot in each experimental condition on both sides, as reported in Table 4 and graphically illustrated in Figure 3. Moreover, load distribution and Regional Contact Area are significantly different across three regions in all conditions on the right side and, on the left one, in the midfoot region with respect to both rearfoot and forefoot, while no significant difference was found between rearfoot and forefoot (Table 4, Figure 3).

In particular, in all conditions, Pmean and Pmax were found to be greater in the rearfoot than forefoot, and in the forefoot than midfoot in both sides. Instead, in all conditions the load distribution and Regional Contact Area were larger in the forefoot than rearfoot, and in the rearfoot than midfoot on the right side (Ff > Rf > Mf), while on the left side, the forefoot and rearfoot did not show significant differences, whereas both of them were greater than the midfoot (Rf ≈ Ff > Mf) (Figure 3).

Regarding the intra-region intra-foot inter-conditions comparison, implemented via repeated-measures ANOVA or Friedman test, the results of Table 5a showed a significant overall difference for the following plantar pressure parameters: rearfoot and forefoot load percentage on both sides; forefoot contact area and mean pressure on both sides; maximum pressure of three regions on the left side and of only the forefoot region on the right side. In particular, the results of post hoc tests corrected for multiple comparisons with the Tukey method (Table 5b) showed significant differences for the following parameters: load %: a significant increase in forefoot load and a significant decrease in rearfoot load on the left side were found in many conditions with HMD-use compared to no-HMD-use ones (HMD-OE vs. OE; HMD-OE vs. CE; HMD-CE vs. OE) and only a significant increase in left forefoot load in the CE condition compared to the OE one; a significant increase in forefoot load and a significant decrease in rearfoot load on the right side were found in all conditions with HMD-use compared to no-HMD-use ones (HMD-OE vs. OE; HMD-OE vs. CE; HMD-CE vs. OE; HMD-CE vs. CE); RCA: a significant increase in left forefoot contact area was found in open and closed eyes conditions with HMD-use compared to OE one (HMD-OE and HMD-CE vs. OE), and in the CE condition compared to the OE one; moreover, a significant increase in right forefoot contact area was found in all conditions with HMD-use compared to no-HMD-use ones, except for the CE condition, only showing a trend; Pmean: a significant increase in forefoot Pmean on both sides in CE and HMD-OE with respect to the OE condition and only on the right side in HMD-CE compared to the OE condition too; Pmax: a significant decrease in left rearfoot maximum pressure in HMD-OE and HMD-CE conditions with respect to the CE one; a significant decrease in left midfoot maximum pressure in HMD–OE with respect to both OE and CE and a significant increase in HMD–CE with respect CE; a significant increase in forefoot maximum pressure on both sides in CE, HMD-OE with respect to the OE condition and only on the right side in HMD-CE compared to OE.

## 4. Discussion

The present study explored the variability of plantar pressure and postural stability parameters in healthy young adults, examining how a standardized immersive environment implemented with virtual reality (VR), as well as the additional mass of a Head-Mounted Display (HMD), may influence these measures.

First, the analysis of intra-subject variability suggested the lowest repeatability for most of the stabilometric parameters, as reported in previous studies [14,20,21,49]. In particular, the highest Coefficient of Variation (CV) was found for Center-of-Pressure sway area (CoPsa) and Length Surface Function (LSF) (30% < median CV < 50%) and a moderate CV for CP speed (10% < median CV < 30%) was found in all visual conditions in both natural and virtual environments (Figure 2). The same analysis applied to plantar pressure parameters confirmed their high repeatability, with the CV below 10% in all conditions, already reported in previous studies [13,14,20,21,49]. No statistically significant differences were found for CVs of all parameters across the four conditions, except for an increase in left midfoot load in the HMD-CE condition compared to the OE one (Table 1). Overall, these findings may indicate that the variability of baropodometric parameters is not substantially influenced by virtual reality. The immersive standardized virtual room did not introduce additional variability, suggesting that VR can be reliably integrated into baropodometric assessments without compromising the consistency of stabilometric or plantar pressure measurements.

As far as the effect of different conditions on stabilometric parameters is concerned, a significant increase in CoPsa and decrease in LSF was found in the closed eyes condition with respect to the open eyes conditions with and without HMD (CE vs. OE; CE vs. HMD-OE). These findings confirmed the typical effect of visual deprivation on postural control, in accordance with previous studies [20,21], suggesting how the lack of visual information remains the predominant factor affecting postural stability.

Within the framework of sensory reweighting, quiet standing depends on the dynamic weighting of visual, vestibular and somatosensory inputs. The increase in CoP sway area during the closed eyes condition is consistent with the reduction in visual contribution and the consequent increased reliance on non-visual afferents. In contrast, the lack of significant differences between OE and HMD-OE suggests that the standardized static virtual room did not induce a detectable destabilizing visual–vestibular conflict in this healthy young sample. The reduction in sway observed in HMD-CE compared with CE should be interpreted cautiously and may be consistent with additional tactile-proprioceptive information provided by the headset or with altered sensory weighting; however, this mechanism cannot be confirmed because HMD mass, contact, occlusion and VR-related factors were not experimentally isolated.

Conversely, this typical effect due to exclusion of visual receptors was not observed when participants wore the Head-Mounted Display, finding no significant difference between the HMD-closed eye condition compared to the open eyes one (HMD-CE vs. OE). This lack of visual-condition-related effects may be explained by the sensory-proprioceptive contribution of wearing the headset itself, which could have compensated for the absence of visual inputs on postural control. This hypothesis may be reinforced by the significant borderline decrease in CoPsa observed in the closed eyes condition while wearing the headset compared with the same condition without the headset (HMD-CE vs. CE); however, this result could be interpreted as a possible effect of the sensory stimulus of the headset on reducing the amplitude of body sway and improving postural stability, in disagreement with previous studies [26,27,45].

Furthermore, no statistically significant difference was found when comparing stabilometric parameters in open eyes conditions with and without the headset (OE vs. HDM-OE) (Table 2). These findings suggest that the standardized static virtual environment did not significantly affect postural stability, in disagreement with [25,26,32,39,40] reporting the effect of virtual environment on stabilometric parameters in healthy adults.

Regarding the effect of different conditions on plantar pressure parameters and foot morphology, it was necessary to evaluate both inter-foot and inter-region intra-foot variations. Specifically, statistical analysis was used to assess significant differences between the left and right foot within each experimental condition, and significant variations across the four conditions were implemented. In addition, the distribution of plantar pressure parameters within the three anatomical regions of the foot (forefoot, midfoot, and rearfoot) and their relationships were examined, considering both within-condition patterns and changes across all experimental conditions. This analytical framework allowed for a comprehensive characterization of foot-specific and condition-dependent variations in plantar pressure parameters, providing a more specific understanding of how visual, virtual reality and Head-Mounted Display inputs modulate pressure-related responses.

Firstly, inter-foot analysis (left vs. right) showed the difference between two feet in the open eyes standard condition and across the other ones for the Arch Index and for total-foot parameters (foot contact area, load percentage, mean and maximum pressures) (Table 3). Results revealed no significant differences between dominant and non-dominant foot for the Arch Index and for total load across all conditions. In contrast, FCA and both mean and maximum pressures displayed significant inter-foot differences (Table 3): specifically, a larger foot contact area was observed on the dominant side (right) than the non-dominant one (left), while a decrease in mean and maximum pressures was found on the dominant side across all conditions (Table 3). The latter result is due to the inverse proportionality between foot contact area and pressure when total load is constant; indeed, for an equivalent load, the wider foot contact area leads to reduced mean pressures (P = F/area). From a neurophysiological and biomechanical point of view, these results may be explained by the attempt of the Postural Control System to maintain symmetrical load distribution between the feet while simultaneously increasing the recruitment area of sensory inputs from the dominant limb. Moreover, these findings related to the comparable distribution of body weight and longitudinal arch morphology between two feet, with a larger foot contact area and lower mean pressure on the right side, which were constantly found unchanged across all conditions, underlining no influence of visual inputs, virtual environments, or proprioceptive stimuli on inter-foot plantar pressure parameters.

Secondly, intra-foot inter-regions analysis of plantar pressure parameters across all conditions revealed the highest mean and peak pressure in the rearfoot, moderate in the forefoot and mild on the midfoot (Table 4 and Table 5a and Figure 3). This inter-regions/intra-foot pressure pattern (Rf > Ff > Mf) remained consistent on both feet across all conditions. Regarding the load distribution and contact area of the three foot regions, two different patterns were found on two feet: the right side showed the highest load percentage and Regional Contact Area in the forefoot, moderate in the rearfoot and mild in the midfoot (Ff > Rf > Mf), while on the left side, the forefoot did not show significant differences with respect to the rearfoot, whereas both the rearfoot and forefoot were larger than the midfoot (Rf ≈ Ff >Mf).

These inter-region intra-foot pressure, contact area and loading patterns persisted across all conditions (Table 4 and Table 5a and Figure 3), confirming the absence of measurable effects attributable to visual receptor, virtual reality exposure and the use of the Head-Mounted Display on inter-region intra-foot relationships.

The observed plantar pressure pattern is consistent with the anatomical and functional organization of quiet bipedal support. The rearfoot and forefoot represent the main load-bearing regions, whereas the midfoot contributes less to vertical loading because of the medial longitudinal arch. Instead, the different pattern (Ff > Rf > Mf right vs. Ff = Rf > Mf left) between two feet, with larger load percentage and Regional Contact Area in the right side than left one, might be explained with the increased total FCA already found in the right dominant foot.

Regarding the effect of different conditions on plantar pressure parameters of each intra-foot region, without considering the relationship among foot regions, the forefoot and rearfoot seem to be influenced by visual condition, HMD use, and the immersive virtual environment, whereas the midfoot did not show significant changes (Table 5). In particular, about body weight distribution on intra-foot regions, an increase in forefoot load percentage and a decrease in the rearfoot one were found on the both sides in HMD-use conditions with respect non-HMD-use ones, and in the closed eyes condition compared to the open eyes one only on the left side. These results suggest an anterior shift of body weight from the rearfoot toward the forefoot related to the use of a headset and to visual deprivation. Consistently, a greater contact area and a higher mean pressure of the forefoot region were found in the abovementioned similar conditions, confirming the specific effects during headset use and the closed eyes condition. These postural adaptations underlined the impact of visual inputs, virtual reality and headset use on plantar pressure parameters of rearfoot and forefoot regions, although the rearfoot-midfoot-forefoot patterns did not show significant changes during different conditions, as previously described. This apparent discrepancy between the increase in the forefoot and unchanged inter-region ratio patterns across all conditions may be explained by significant intra-regional variations in the foot, but not such as to alter the relationship between the different regions. These findings should be interpreted by distinguishing absolute regional values from relative intra-foot distribution patterns. Although some forefoot and rearfoot parameters changed across conditions, the overall rearfoot-midfoot-forefoot hierarchy remained substantially preserved. Thus, HMD-based testing and visual deprivation were associated with localized modifications in regional plantar loading but did not markedly alter the global intra-foot distribution pattern observed in quiet standing.

In summary, in the investigated young adult population of the present study, the plantar pressure parameters highlighted the following specific features on two feet in the standardized open eyes condition: a similar total load percentage between two sides, with a larger foot contact area and lower total mean pressure on the dominant side. Moreover, two different load percentage and Regional Contact Area patterns were found among the three foot regions: Ff > Rf > Mf pattern on the dominant side and Rf ≈ Ff > Mf pattern on the non-dominant one. Instead, a fixed pressure pattern was found on two feet: Rf > Ff > Mf. All these plantar pressure inter-foot and intra-foot inter-region ratio patterns never changed across all conditions, whereas significant changes in intra-foot region were found in the rearfoot and forefoot during headset use and visual deprivation.

Eventually, regarding postural stability assessment, the visual deprivation remains the main factor influencing postural stability and increasing body sway area; conversely, VR does not cause changes, while the headset use can reduce body sway area probably due to sensory-proprioceptive inputs affecting postural control. Finally, the use of VR seems not to increase the variability of postural stability and plantar pressure parameters.

For all these reasons, administering an immersive and standardized virtual room through an HMD appears to be a valuable tool for postural assessment, especially for ensuring consistent testing and controlled environments. The use of VR enables the reproduction of identical visual conditions across participants, even when physical testing space is limited, without introducing additional variability into stabilometric measurements.

This study presents the following limitations: unbalanced sex distribution of the sample and young population; possible undiagnosed dental malocclusion which could potentially affect postural control. Moreover, the results are specific to the weight and size of the headset used in this study and cannot be generalized to other different headsets.

Another limitation of the study is that the experimental design did not allow a clear distinction between the effects related to the virtual reality environment and those caused by wearing the Head-Mounted Display itself. Some additional limitations of the statistical approach should be acknowledged. First, intra-subject variability was estimated by CV over only three trials per condition; although this procedure was applied consistently across all conditions, such estimates should be interpreted with caution. Second, the analysis of plantar pressure variables required multiple related comparisons across sides, regions, and conditions; despite the use of corrected post hoc procedures within each analytical family, these findings should be interpreted as partially exploratory. Finally, effect size estimates would further strengthen the interpretation of the practical magnitude of the observed differences.

Future studies should recruit a larger and age-heterogeneous population, and consider the effect of other sensory afferents, such as stomatognathic ones, which can affect postural control.

## 5. Conclusions

The present study showed the effect of virtual reality and headset use on plantar pressure and postural stability parameters. In particular, no impact on variability was observed, underlining a good repeatability of the abovementioned parameters during baropodometric exam with VR headset use. Moreover, no effect of virtual environment on postural stability was found, while a specific sensory-proprioceptive effect emerged when wearing a Head-Mounted Display in the closed eyes condition. Finally, plantar pressure parameters between the two feet and among the foot regions did not show significant changes across conditions, except for an increase in forefoot load percentage, mean pressure and contact area during VR headset use and visual deprivation, without modifying the inter-regional area ratio. These results suggest the possibility of creating a standardized setting for baropodometric assessments using VR, identically reproducible across sites without affecting the variability of plantar pressure and postural stability parameters related to differences among laboratory environments. Moreover, the immersive VR environment offers the opportunity of generating comparable datasets across research centers and the potential flexibility of environmental features, which can be adjusted in relation to specific requirements of a clinical assessment or rehabilitation protocol.

## Figures and Tables

**Table 1:** (a) General statistical analysis implemented via repeated-measures ANOVA or Friedman test across four conditions for CV of parameters resulted in significant differences (CVs of left load midfoot). (b) Post hoc test, corrected with Tukey method. *p*-value with other details of the implemented test were reported (F or χ^2^; η^2^_p_ or W; and df that are statistic, effect size and degree of freedom respectively, for ANOVA and Friedman).

**(a)**							
**CV of Parameters**	**LS**	**OE**	**CE**	**HMD-OE**	**HMD-CE**	**Repeated-Measures ANOVA** **or** **Friedman Test**	
		**Mean ± SD (Min; Max) or Median (25th–75th Percentiles)**				***p *** **(F ** **or ** **χ^2^; η^2^_p_ or W; df)**	
**Load Mf [%]**	**l**	7.83 (3.86; 19.44)	7.98 (4.23; 16.39)	7.35 (3.75; 19.64)	13.92 (4.78; 22.74)	**0.044 (8.09; 0.054; 3)**	
**(b)**							
**CV of Parameters**	**LS**	**OE vs. CE**	**HMD-OE vs.** **HMD-CE**	**OE vs.** **HMD-OE**	**CE vs. HMD-CE**	**OE vs. HMD-CE**	**CE vs. HMD-OE**
**Load Mf [%]**	**l**	0.582 (0.55)	0.582 (0.55)	0.051 (1.97)	0.051 (1.97)	**0.013 (2.25)**	0.158 (1.42)

Notes: Limb Side (LS), left (l), right (r), midfoot (Mf), Open Eyes (OE); Closed Eyes (CE), virtual reality (VR), Head-Mounted Display (HMD), Coefficient of Variation (CV), *p*-Value (*p*).

**Table 2:** Inter-condition multiple comparisons for stabilometric parameters. (a) General statistical analysis implemented via repeated-measures ANOVA or Friedman test. (b) Post hoc test, corrected with Tukey method, for parameters resulted in significant differences (CoPsa and LSF) via repeated-measures ANOVA or Friedman test; *p*-value (≤0.05) with other details of the implemented test were reported (F or χ^2^; η^2^_p_ or W; and df that are statistic, effect size and degree of freedom respectively, for ANOVA and Friedman).

**(a)**						
**Stabilometric** **Parameters**	**OE**	**CE**	**HMD-OE**	**HMD-CE**	**Repeated-Measures ANOVA** **or Friedman Test**	
	**Mean ± SD (Min; Max) or Median (25th–75th Percentiles)**				***p *** **(F or ** **χ^2^; η^2^_p_ or W; df)**	
**CoPsa [mm^2^]**	29.9 (19.6; 57.2)	42.1 (29.2; 72.1)	32.9 (19.3; 62.5)	33.7 (19.8; 55.2)	**0.029 (9.05; 0.060; 3)**	
**CP speed [mm/s]**	4.92 ± 1.20 (2.70; 7.83)	5.02 ± 1.15 (2.23; 7.43)	4.74 ± 1.05 (2.23; 7.43)	4.89 ± 1.09 (2.70; 7.67)	0.168 (1.78; 0.008; 2.33)	
**LSF [mm ^−1^ ]**	5.53 (3.65; 8.08)	4.02 (2.67; 6.44)	5.53 (3.37; 7.97)	5.03 (3.51; 7.60)	**0.013 (10.7; 0.071; 3)**	
**(b)**						
**Stabilometric** **Parameters**	**OE vs. CE**	**HMD-OE vs. HMD-CE**	**OE vs. HMD-OE**	**CE vs. HMD-CE**	**OE vs. HMD-CE**	**CE vs. HMD-OE**
	***p *(t or *χ*^2^)**					
**CoPsa [mm^2^]**	**0.008 (2.69)**	0.528 (0.63)	0.937 (0.08)	**0.050** (1.98)	0.478 (0.71)	**0.010 (2.61)**
**LSF [mm ^−1^ ]**	**0.003 (3.02)**	0.341 (0.96)	0.812 (0.24)	0.069 (1.83)	0.235 (1.19)	**0.006 (2.785)**

Notes: Center of Pressure (CP), Center-of-Pressure sway area (CoPsa), Open Eyes (OE); Closed Eyes (CE), virtual reality (VR), Head-Mounted Display (HMD), *p*-Value (*p*).

**Table 3:** Inter-foot analysis in each condition and inter-conditions analysis for each foot of load Total foot, foot contact area and Arch Index implemented via two sides repeated-measures ANOVA. *p*-values (≤0.05) with statistics of the implemented test were reported (t for inter sides comparison; while F or χ^2^ for inter condition comparison).

Plantar Pressure Parameter	LS	OE		CE		HMD-OE		HMD-CE		Repeated-Measures ANOVA or Friedman Test
		Mean ± SD (Min; Max) or Median (25th–75th Percentiles)	*p* (*t*) Inter Sides (Right vs. Left)	Mean ± SD (Min; Max) or Median (25th–75th Percentiles)	*p* (F or χ^2^) Inter Sides (Right vs. Left)	Mean ± SD (Min; Max) or Median (25th–75th Percentiles)	*p* (F or χ^2^) Inter Sides (Right vs. Left)	Mean ± SD (Min; Max) or Median (25th–75th Percentiles)	*p* (F or χ^2^) Inter-Sides (Right vs. Left)	*p* (F or χ^2^) Inter Conditions
**AI** **[%]**	**l**	10.12 (4.66; 18.7)	0.791 (−1.52)	9.00 (4.61; 18.2)	0.715 (1.66)	9.47 (4.86; 18.5)	0.708 (−1.67)	7.91 (4.78; 18.8)	0.795 (−1.52)	0.455 (2.62)
	**r**	9.89 (5.37, 19.9)		10.94 (5.74; 19.6)		10.90 (6.24; 18.8)		9.97 (5.67; 20.00)		0.483 (2.46)
**FCA** **[cm ^2^ ]**	**l**	102 ± 25.5 (64.3; 162)	**<0.001** **(−6.88)**	103 ± 24.8 (61.0; 165)	**<0.001** **(−5.67)**	104 ± 22.7 (68.0; 154)	**<0.001** **(−5.50)**	104 ± 22.7 (68.0; 154)	**<0.001** **(5.39)**	0.366 (0.983)
	**r**	110 ± 28.3 (67.7; 150)		111 ± 27.8 (69.0; 179)		112 ± 25.8 (65.3; 169)		112 ± 26.5 (68.0; 161)		0.120 (5.84)
**Pmean Tf [kPa]**	**l**	34.4 ± 5.76 (22.9; 44.9)	**0.005** **(3.98)**	33.9 ± 5.48 (22.6; 43.9)	**0.011** **(3.74)**	33.4 ± 4.61 (24.1; 42.1)	**0.009** **(3.78)**	33.8 ± 5.34 (22.9; 49.7)	0.066 (3.05)	0.129 (2.04)
	**r**	33.1 ± 4.88 (23.1; 41.4)		32.7 ± 4.55 (22.7; 40.7)		32.3 ± 4.07 (24.2; 41.7)		32.3 ± 4.19 (23.4; 41.3)		0.067 (2.99)
**Pmax Tf [kPa]**	**l**	88.3 ± 14.4 (54.8; 128)	**0.003** **(4.14)**	87.5 ± 13.8 (53.3; 123)	**0.014** **(3.63)**	86.5 ± 12.7 (59.9; 126)	**0.004** **(4.07)**	86.4 ± 12.6 (58.2; 125)	**0.002** **(4.35)**	0.117 (2.27)
	**r**	83.4 ± 15.1 (56.4; 116)		83.0 ± 14.0 (54.6; 112)		81.2 ± 12.4 (59.0; 117)		81.6 ± 12.4 (58.5; 116)		0.192 (2.027)
**Load Tf** **[%]**	**l**	49.1 ± 2.15 (43.1; 55.2)	0.093 (−2.91)	49.1 ± 2.33 (43.4; 55.5)	0.136 (−2.74)	49 ± 2.90 (42.9; 57.3)	0.256 (−2.42)	48.9 ± 2.81 (43.3; 56.3)	0.161 (−2.66)	0.842 (0.277)
	**r**	50.9 ± 2.15 (44.8; 56.9)		50.9 ± 2.33 (44.5; 56.6)		51.0 ± 2.90 (42.7; 57.1)		51.1 ± 2.81 (43.7; 56.6)		0.842 (0.277)

Notes: Standard Deviation (SD), mean pressure (Pmean), maximum pressure (Pmax), Limb Side (LS), left (l), right (r), Total foot (Tf), Foot Contact Area (FCA), Arch Index (AI), Open Eyes (OE); Closed Eyes (CE), virtual reality (VR), Head-Mounted Display (HMD), *p*-value (*p*).

**Table 4:** Intra-foot and inter-region multiple comparisons for load, Pmax and Pmean were implemented via non parametric Friedman test across three foot regions in each condition (OE, CE, HMD-OE and HMD-CE). *p*-values (≤0.05) with other details of the implemented test were reported (χ^2^; W; and df that are statistic, effect size and degree of freedom respectively, for Friedman test).

Condition	Plantar Pressure Parameter	Left Side				Right Side			
		Repeated-Measures ANOVA or Friedman Test	Post Hoc			Repeated-Measures ANOVA or Friedman Test	Post Hoc		
			Rf vs. Ff	Mf vs. Ff	Mf vs. RF		Rf vs. Ff	Mf vs. Ff	Mf vs. RF
		*p* (χ^2^; W; df)	*p* (t or χ^2^)			*p* (χ^2^; W; df)	*p* (t or χ^2^)		
**OE**	**Load [%]**	**<0.001** **(66.30; 66.3; 2)**	**0.865** **(0.17)**	**<0.001** **(11.993)**	**<0.001** **(12.104)**	**<0.001** **(71.10; 0.711; 2)**	**0.011** **(2.58)**	**<0.001** **(14.54)**	**<0.001** **(11.97)**
	**Pmax [kPa]**	**<0.001** **(75.60; 0.756; 2)**	**<0.001** **(12.84)**	**<0.001** **(3.81)**	**<0.001** **(16.65)**	**<0.001** **(75.00; 0.750; 2)**	**<0.001** **(10.30)**	**<0.001 (6.74)**	**<0.001** **(17.04)**
	**Pmean [kPa]**	**<0.001** **(88.80; 0.888; 2)**	**<0.001** **(15.70)**	**<0.001** **(12.10)**	**<0.001** **(27.90)**	**<0.001** **(81.10; 0.811; 2)**	**<0.001** **(10.94)**	**<0.001** **(9.57)**	**<0.001** **(20.50)**
	**RCA [cm^2^]**	**<0.001** **(66.30; 0.663; 2)**	0.865 (0.17)	**<0.001** **(12.10)**	**<0.001** **(11.93)**	**<0.001** **(71.70; 0.717; 2)**	**0.004** **(2.98)**	**<0.001** **(14.88)**	**<0.001** **(11.91)**
**CE**	**Load [%]**	**<0.001** **(64.40; 0.664; 2)**	0.610 (0.512)	**<0.001** **(12.29)**	**<0.001** **(11.78)**	**<0.001** **(69.20; 0.682; 2)**	**0.003** **(3.03)**	**<0.001** **(14.08)**	**<0.001** **(11.05)**
	**Pmax [kPa]**	**<0.001** **(78.20; 0.782; 2)**	**<0.001** **(13.07)**	**<0.001** **(5.10)**	**<0.001** **(18.17)**	**<0.001** **(78.50; 0.785; 2)**	**<0.001** **(11.32)**	**<0.001** **(7.48)**	**<0.001** **(18.80)**
	**Pmean [kPa]**	**<0.001** **(85.70; 0.857; 2)**	**<0.001** **(14.41)**	**<0.001** **(9.69)**	**<0.001** **(24.10)**	**<0.001** **(86.50; 0.865; 2)**	**<0.001** **(12.90)**	**<0.001** **(12.10)**	**<0.001** **(25.10)**
	**RCA [cm^2^]**	**<0.001** **(66.30; 0.663; 2)**	0.865 (0.17)	**<0.001** **(12.10)**	**<0.001** **(11.93)**	**<0.001** **(70.7; 0.707; 2)**	**<0.001** **(3.84)**	**<0.001** **(14.81)**	**<0.001** **(10.97)**
**HMD-OE**	**Load [%]**	**<0.001** **(71.30; 0.713; 2)**	0.231 (1.21)	**<0.001** **(14.09)**	**<0.001** **(12.89)**	**<0.001** **(80.40; 0.804; 2)**	**<0.001** **(6.49)**	**<0.001** **(19.70)**	**<0.001** **(13.21)**
	**Pmax [kPa]**	**<0.001** **(82.70; 0.827; 2)**	**<0.001** **(14.68)**	**<0.001** **(6.44)**	**<0.001** **(21.12)**	**<0.001** **(85.70; 0.857; 2)**	**<0.001** **(14.41)**	**<0.001** **(6.69)**	**<0.001** **(24.10)**
	**Pmean [kPa]**	**<0.001** **(91.00;0.910; 2)**	**<0.001** **(18.10)**	**<0.001** **(13.20)**	**<0.001** **(31.30)**	**<0.001** **(92.20; 0.922; 2)**	**<0.001** **(17.00)**	**<0.001** **(17.00)**	**<0.001** **(33.90)**
	**RCA [cm^2^]**	**<0.001** **(69.50; 0.695; 2)**	0.285 (1.08)	**<0.001** **(13.44)**	**<0.001** **(12.36)**	**<0.001** **(84.30; 0.843; 2)**	**<0.001** **(8.74)**	**<0.001** **(22.72)**	**<0.001** **(13.98)**
**HMD-CE**	**Load [%]**	**<0.001** **(69.20; 0.692; 2)**	0.722 (0.36)	**<0.001** **(13.01)**	**<0.001** **(12.66)**	**<0.001** **(77.00; 0.770; 2)**	**<0.001** **(5.77)**	**<0.001** **(17.74)**	**<0.001** **(11.96)**
	**Pmax [kPa]**	**<0.001** **(81.60; 0.816; 2)**	**<0.001** **(13.40)**	**<0.001** **(7.16)**	**<0.001** **(20.56)**	**<0.001** **(85.70; 0.857; 2)**	**<0.001** **(14.41)**	**<0.001** **(9.69)**	**<0.001** **(24.10)**
	**Pmean [kPa]**	**<0.001** **(91.00; 0.910; 2)**	**<0.001** **(18.10)**	**<0.001** **(13.20)**	**<0.001** **(31.30)**	**<0.001** **(90.30; 0.903; 2)**	**<0.001** **(15.60)**	**<0.001** **(14.60)**	**<0.001** **(30.20)**
	**RCA [cm^2^]**	**<0.001** **(69.50; 0.695; 2)**	0.285 (1.08)	**<0.001** **(13.44)**	**<0.001** **(12.36)**	**<0.001** **(82.90; 0.829; 2)**	**<0.001** **(7.90)**	**<0.001** **(21.56)**	**<0.001** **(13.65)**

Notes: Significant differences are in bold (*p*-value ≤ 0.05); rearfoot (Rf), midfoot (Mf), forefoot (Ff), mean pressure (Pmean), maximum pressure (Pmax), Regional Contact Area (RCA), Open Eyes (OE); Closed Eyes (CE), virtual reality (VR), Head-Mounted Display (HMD), *p*-value (*p*).

**Table 5:** Inter-condition multiple comparisons for plantar pressure parameters (load, Pmean and Pmax) of each region of the foot on both sides. (a) Mean or median values and a significant overall difference among four conditions implemented via repeated-measures ANOVA or Friedman test. (b) Post hoc test, corrected with Tukey method, for parameters resulted in significant differences (load and Pmax of Rf and Ff, Pmax of Mf and Pmean of Ff). *p*-values (≤0.05) with other details of the implemented test were reported (F or χ^2^; η^2^_p_ or W; and df that are statistic, effect size and degree of freedom respectively, for ANOVA and Friedman).

**(a)**							
**Plantar Pressure** **Parameter**	**LS**	**OE**	**CE**	**HMD-OE**	**HMD-CE**	**Repeated-Measures ANOVA** **or Friedman Test**	
		**Mean ± SD (Min; Max) or Median (25th–75th Percentiles)**				***p *** **(F or χ^2^; η^2^_p_ or W; df)**	
**Load Rf [%]**	**l**	45.6 ± 7.76 (32.63; 61.8)	44.8 ± 7.16 (32.30; 59.7)	44.2 ± 6.77 (32.73; 61.3)	44.3 ± 6.61 (33.13; 57.1)	**0.002 (6.27; 0.006; 2.35)**	
	**r**	42.7 ± 7.66 (30.93; 60.3)	42.3 ± 7.12 (31.17; 59.7)	41.5 ± 6.59 (30.0; 61.7)	41.3 ± 6.59 (25.13; 59.8)	**0.001 (6.48; 0.007; 2.28)**	
**Load Mf [%]**	**l**	10.12 (4.67; 18.7)	9.00 (4.59; 18.2)	9.47 (4.86; 18.5)	7.92 (4.78; 18.8)	0.500 (2.37; 0.016; 3)	
	**r**	9.88 (5.39; 20.0)	10.92 (5.75; 19.6)	10.92 (6.24; 18.8)	9.98 (5.65; 19.6)	0.520 (2.26; 0.015; 3)	
**Load Ff [%]**	**l**	42.3 ± 7.92 (16.93; 58.6)	43.2 ± 7.19 (19.23; 58.3)	43.50 ± 7.19 (19.23; 58.3)	44.48 ± 6.87 (18.97; 59.4)	**<0.001 (8.38; 0.008; 2.27)**	
	**r**	44.1 ± 7.73 (16.23; 60.7)	44.6 ± 6.65 (24.7; 59.1)	45.5 ± 6.50 (23.67; 59.9)	45.9 ± 6.41 (25.87; 59.3)	**<0.001 (11.0; 0.012; 2.64)**	
**Pmean Rf [kPa]**	**l**	50.1 ± 9.56 (25.43; 71.0)	50.4 ± 9.39 (25.21; 68.4)	49.3 ± 7.97 (34.77; 71.6)	49.3 ± 8.57 (30.55; 70.2)	0.110 (2.20; 0.003; 2.22)	
	**r**	47.4 ± 9.68 (28.41; 66.0)	47.7 ± 9.53 (29.12; 68.0)	46.7 ± 8.28 (30.28; 66.4)	46.8 ± 8.80 (30.18, 72.0)	0.254 (1.39; 0.002; 2.04)	
**Pmean Mf [kPa]**	**l**	20.0 (17.2; 25)	20.4 (18.3; 24.0)	20.3 ± 4.38 (12.64; 33.9)	20.2 ± 5.54 (6.76; 35.5)	0.079 (6.79; 0.045; 3)	
	**r**	20.6 (17.2; 23.9)	20.5 (17.3, 23.4)	19.4 (17.5; 22.8)	20.4 ± 4.45 (10.16; 32.2)	0.159 (5.18; 0.035; 3)	
**Pmean Ff [kPa]**	**l**	28.3 ± 4.89 (15.38; 44.2)	28.7 ± 4.97 (17.61; 44.3)	28.8 ± 4.71 (19.77; 44.4)	28.4 ± 4.69 (17.42; 43.2)	**0.015 (10.5; 0.070; 3)**	
	**r**	29.9 ± 5.24 (19.45; 47.4)	30.5 ± 4.75 (22.40; 44.7)	30.7 ± 4.68 (20.75; 44.3)	30.5 ± 4.95 (22.19; 44.6)	**0.010 (11.4; 0.076; 3)**	
**RCA Rf [cm^2^]**	**l**	42.4 ± 5.58 (31.37; 54.2)	42.3 ± 5.86 (31.03; 58.0)	42.1 ± 5.21 (32.9; 56.9)	42.0 ± 5.33 (30.83; 58.7)	0.393 (1.00; 0.001; 3)	
	**r**	42.0 ± 6.16 (32.37; 53.9)	42.4 ± 6.31 (32.17; 56.5)	42 ± 6.30 (32.17; 58.6)	41.9 ± 6.55 (30.87; 59.8)	0.233 (1.47; 0.001; 2.14)	
**RCA Mf [cm^2^]**	**l**	8.22 (3.95; 19.0)	10.17 (3.68; 19.6)	9.15 (3.69; 18.1)	6.53 (4.03; 18.8)	0.583 (1.95; 0.013; 3)	
	**r**	9.30 (4.90; 21.2)	9.82 (5.44; 20.9)	11.67 (5.74; 22.9)	10.68 (5.16; 22.0)	0.644 (1.67; 0.011; 3)	
**RCA Ff [cm^2^]**	**l**	43.0 ± 15.51 (13.8; 90.8)	44.5 ±14.66 (18.07; 88.90)	45.5 ± 13.99 (15.88; 85.8)	45.2 ± 13.7 (16.60; 82.9)	**0.013 (4.67; 0.005; 1.88)**	
	**r**	48.2 ± 16.99 (13.4;105.6)	49.2 ± 16.11 (21.73; 101.3)	50.8 ± 15.10 (24.20; 99.10)	51.4 ± 15.91 (21.0; 93.2)	**0.001(7.22; 0.006; 2.06)**	
**Pmax Rf [kPa]**	**l**	94.8 ± 16.44 (51.7; 140)	95.6 ± 16.91 (52.7; 144.7)	93.5 ± 14.53 (65.0; 141)	93.1 ± 14.47 (62.3; 133.7)	**0.028 (3.71; 0.004; 2.01)**	
	**r**	89.5 ± 16.93 (59.0; 125.3)	90.6 ± 17.08 (59.0; 129.3)	88.6 ± 15.12 (60.7; 130.3)	87.8 ± 14.50 (56.7; 123)	0.064 (2.96; 0.004; 1.75)	
**Pmax Mf [kPa]**	**l**	36.3 (20.4; 45.6)	36.8 (28.5; 44.8)	36.1 ± 11.31 (16.7; 74.0)	37.5 (27.9, 43.8)	**0.010 (11.3; 0.075; 3)**	
	**r**	34.8 (29.1; 40.6)	36.7 (30.0; 42.6)	35.2 (29.1; 39.2)	34.2 (28.0; 39.7)	0.154 (5.25; 0.035; 3)	
**Pmax Ff [kPa]**	**l**	48.3 ± 10.68 (24.0; 76.3)	49.7 ± 10.52 (28.3; 74.0)	49.8 ± 10.36 (28.3; 74.0)	49.3 ± 10.43 (26.0; 72.7)	**0.024 (3.74; 0.003; 2.17)**	
	**r**	51.2 ± 11.24 (26.0; 79.0)	52.9 ± 10.42 (33.0; 77.0;)	53.3 ± 10.23 (32.7; 75.7)	52.9 ± 9.94 (36.0; 74.7)	**0.006 (5.36; 0.006; 2.04)**	
**(b)**							
**Plantar Pressure** **Parameter**	**LS**	**OE vs. CE**	**HMD-OE vs. HMD-CE**	**OE vs. HMD-OE**	**CE vs. HMD-CE**	**OE vs. HMD-CE**	**CE vs. HMD-OE**
		***p *** **(t or *χ*^2^)**					
**Load Rf [%]**	**l**	**0.013 (2.58)**	0.598 (0.53)	**<0.001 (3.55)**	0.196 (1.31)	**0.007 (2.80)**	0.061 (1.92)
	**r**	0.107 (1.64)	0.696 (0.39)	**0.002 (3.34)**	**0.027 (2.28)**	**0.003 (3.08)**	**0.021 (2.39)**
**Load Ff [%]**	**l**	**0.006 (2.90)**	0.616 (0.51)	**<0.001 (3.76)**	0.084 (1.76)	**0.002 (3.31)**	**0.020 (2.40)**
	**r**	0.102 (1.67)	0.235 (1.20)	**<0.001 (3.59)**	**<0.001 (3.71)**	**<0.001 (4.45)**	**0.007 (2.82)**
**Pmean Ff [kPa]**	**l**	**0.008 (2.70)**	0.114 (1.59)	**0.004 (2.94)**	0.178 (1.35)	0.178 (1.35)	0.812 (0.24)
	**r**	**0.005 (2.88)**	0.426 (0.80)	**0.002 (3.11)**	0.577 (0.56)	**0.022 (2.31)**	0.811 (0.24)
**RCA Ff [cm^2^]**	**l**	**0.023 (2.97)**	0.945 (0.55)	**0.020 (3.03)**	0.832 (0.85)	**0.103 (2.34)**	0.513 (1.39)
	**r**	0.285(1.80)	0.835 (0.84)	**0.007 (3.41)**	0.075 (2.48)	**0.013 (3.17)**	**0.043 (2.72)**
**Pmax Rf [kPa]**	**l**	0.191 (1.33)	0.510 (0.66)	0.147 (1.48)	**0.023 (2.34)**	0.082 (1.78)	**0.021 (2.38)**
**Pmax Mf [kPa]**	**l**	0.442 (0.771)	0.241 (1.176)	**0.016 (2.434)**	**0.044 (2.028)**	0.211 (1.257)	**0.002 (3.204)**
**Pmax Ff [kPa]**	**l**	**<0.001 (3.70)**	0.202 (1.29)	**0.014 (2.54)**	0.451 (0.76)	0.075 (1.82)	0.891 (0.14)
	**r**	**<0.001 (3.82)**	0.430 (0.80)	**0.006 (2.86)**	0.940 (0.08)	**0.008 (2.79)**	0.555 (0.60)

Notes: Limb Side (LS), left (l), right (r), Total foot (Tf), rearfoot (Rf), midfoot (Mf), forefoot (Ff), mean pressure (Pmean), maximum pressure (Pmax), Regional Contact Area (RCA), Open Eyes (OE); Closed Eyes (CE), virtual reality (VR), Head-Mounted Display (HMD), *p*-value (*p*).

## Data Availability

The datasets analyzed during the current study are available from the corresponding author on reasonable request.

## Abbreviations

The following abbreviations are used in this manuscript:

VR	virtual reality
HMD	Head-Mounted Display
OE	Open Eyes
CE	Closed Eyes
HMD-OE	Head-Mounted Display with Open Eyes
HMD-CE	Head-Mounted Display with Closed Eyes
CNS	Central Nervous System
PCS	Postural Control System
RQ	Research Question
ANOVA	Analysis of Variance
BMI	Body Mass Index
CoPsa	Center-of-Pressure sway area
LSF	Length Surface Function
CP-speed	Center-of-Pressure speed
Tf	Total foot
Rf	rearfoot
Mf	midfoot
Ff	forefoot
FCA	Foot Contact Area
AI	Arch Index
Pmean	mean pressure
Pmax	maximum pressure
RCA	Regional Contact Area
CV	Coefficient of Variation
SD	Standard Deviation
LS	Limb Side
CoP	Center of Pressure
